# The role of the deubiquitinating enzyme DUB3/USP17 in cancer: a narrative review

**DOI:** 10.1186/s12935-021-02160-y

**Published:** 2021-08-28

**Authors:** Guang-Fei Yang, Xin Zhang, Yi-Ge Su, Ren Zhao, Yan-Yang Wang

**Affiliations:** 1 Dept. of Ultrasound, General Hospital of Ningxia Medical University, Yinchuan, 750004 Ningxia China; 2 School of Clinical Medicine, Ningxia Medical University, Yinchuan, 750004 Ningxia China; 3 Graduate School, Ningxia Medical University, Yinchuan, 750004 Ningxia China; 4 Dept. of Radiation Oncology, General Hospital of Ningxia Medical University, Yinchuan, 750004 Ningxia China; 5 Cancer Institute, Ningxia Medical University, Yinchuan, 750004 Ningxia China

**Keywords:** DUB3**/**USP17, Cancer, Ubiquitination, Deubiquitinating enzymes, Signaling pathways

## Abstract

The balance between ubiquitination and deubiquitination is critical for the degradation, transport, localization, and activity of proteins. Deubiquitinating enzymes (DUBs) greatly contribute to the balance of ubiquitination and deubiquitination, and they have been widely studied due to their fundamental role in cancer. DUB3/ubiquitin-specific protease 17 (USP17) is a type of DUB that has attracted much attention in cancer research. In this review, we summarize the biological functions and regulatory mechanisms of USP17 in central nervous system, head and neck, thoracic, breast, gastrointestinal, genitourinary, and gynecologic cancers as well as bone and soft tissue sarcomas, and we provide new insights into how USP17 can be used in the management of cancer.

## Introduction

Protein is the basic unit that regulates many cellular processes in eukaryotic cells. Ubiquitination is the second most common posttranslational modification (PTM), and it modifies the activity, localization, interaction, and stability of proteins [1]. Furthermore, ubiquitination also affects many other cellular and biological processes, such as cell cycle control, DNA repair, transcriptional regulation, immune response, and apoptosis [2]. Therefore, abnormal ubiquitination may lead to various diseases, including cancer [3, 4].

The ubiquitination process is achieved by the following three types of enzymes: ubiquitin-activating enzyme (E1), ubiquitin-conjugating enzyme (E2), and ubiquitin ligase (E3) [5]. The ubiquitination process can also be reversed by deubiquitinating enzymes (DUBs) [6], which affect various signaling pathways by removing ubiquitin from substrates [7]. Ubiquitin-specific proteases (USPs) account for the largest proportion of DUBs in the human genome with nearly 70 members [8]. In recent years, the oncogenic or tumor suppressive function of USPs has been assessed in many studies [9], and all studies have demonstrated the potential role of USPs against cancer.

USP17, also referred to as DUB3, is regulated by interleukin-4 (IL-4) and IL-6 cytokines [10]. The abnormal expression of USP17 is related to inflammation, cell motility, the development of T helper 17 (Th17) cells, and carcinogenesis [11]. Several studies have found that USP17 plays a critical role in the carcinogenesis and progression of different types of cancer, but an in-depth review on the role of USP17 in cancers is lacking. Therefore, this review summarized the biological functions and regulatory mechanisms of USP17 in various types of cancer to provide new possibilities for the management of cancers.

## Ubiquitination process

Ubiquitination refers to the process of covalent conjugation of ubiquitin, which is a protein composed of 76 amino acids that attach to a lysine residue on substrate proteins. The process requires the following three types of enzymes: E1, E2, and E3. In humans, there are 2 E1 enzymes, approximately 50 E2 enzymes, and approximately 600 E3 ubiquitin ligases [12].

In the first step, E1 hydrolyzes adenosine triphosphate (ATP) and catalyzes the C-terminal acyl adenylation of ubiquitin. Ubiquitin is then transferred to the cysteine residue of the active site of E1, which is accompanied by the adenylation of the second ubiquitin. The adenylated ubiquitin is then transferred to the cysteine residue in the ubiquitin conjugation domain of E2. The final step of conjugating ubiquitin to the target is mediated by E3 ubiquitin ligases. E3 is responsible for substrate identification and transfer, and it plays a key role in determining the specific type of ubiquitinated substrate [13, 14].

According to the different catalytic structures, E3 enzymes can be divided into three families as follows: really interesting new gene (RING), homologous to the E6-AP carboxyl terminus (HECT), and the ring between ring fingers (RBR) family [15]. RING is the largest family of E3 ligases that help transfer ubiquitin connected to E2 directly to the substrate without forming a thioester bond with ubiquitin [16, 17]. The amount of HECT E3 is less than that of RING E3. HECT E3 forms catalytic cysteine-dependent intermediates with ubiquitin linked to E2, which are then transferred to the target protein [18]. RBR is a special type of E3 ligase, and its activation mechanism is different from that of RING and HECT. RBR has two RING domains (RING1 and RING2) and a domain between the RING domains (IBR). The RING1 domain initially recognizes the ubiquitin attached to E2, and the RING2 domain then provides a cysteine residue to the active site of E2, which forms a thioester-linked E2-ubiquitin intermediate [19, 20].

Ubiquitination can be divided into polyubiquitination and monoubiquitination based on the number of ubiquitins attached to proteins [21]. Different types of ubiquitination modifications are related to different physiological functions in cells. Usually, the polyubiquitination process is involved in both protein degradation and signal transduction. For example, Lys-48(K48)-branched polyubiquitination regulates protein stability through 26S proteasome-mediated degradation. The K63-linked polyubiquitination chain executes a nonproteasome process. However, monoubiquitination is involved in the regulation of diverse cellular processes, such as DNA repair, signal transduction, receptor endocytosis, and stress response [22, 23].

**Fig. 1:**
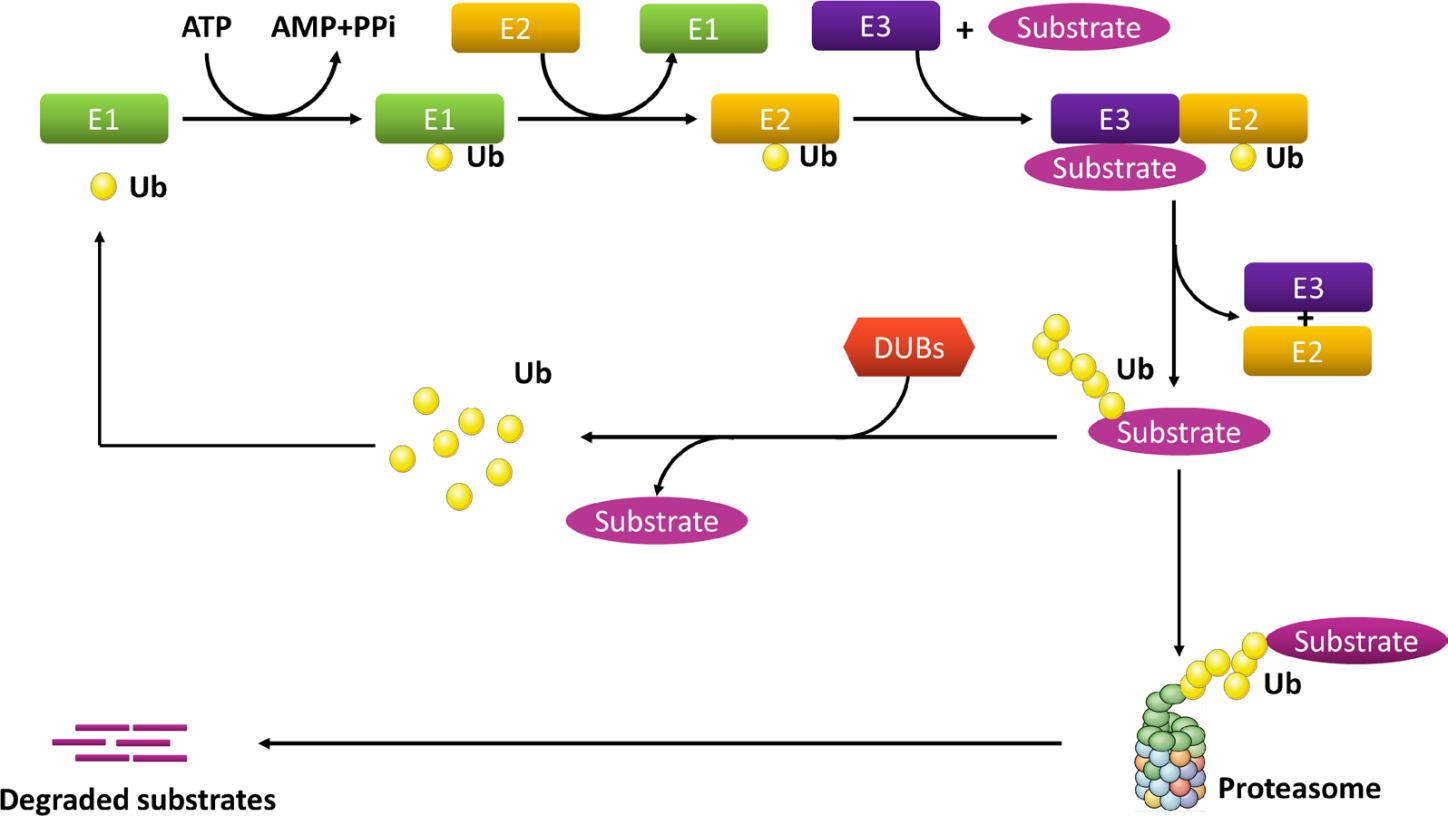
The ubiquitination process. E1 hydrolyzes adenosine triphosphate (ATP) to catalyze the C-terminal acyl adenylation of ubiquitin. Then, ubiquitin is transferred to the cysteine residue of E1 active site, accompanied by the adenylation of the second ubiquitin. The adenylated ubiquitin is then transferred to the cysteine residue of the ubiquitin conjugated domain of E2. The final step in the binding of ubiquitin to the target is mediated by E3 ubiquitin ligase. The ubiquitinated proteins are then recognized and degraded by the proteasome. Ubiquitinated proteins can also be deubiquitinated by deubiquitin enzyme (DUBs)

The ubiquitination process can be reversed by DUBs, which belong to the family of cysteine proteases. DUBs cleave the isopeptide bond or the peptide bond with high specificity, and they are responsible for removing ubiquitin from the substrate and maintaining its stabilization [24]. DUBs can be divided into the following six families according to sequence and structure: ubiquitin-specific proteases (USPs), ubiquitin COOH terminal hydrolases (UCHs), ovarian tumor proteases (OTUs), Machado–Joseph (Josephin) domain proteases (MJDs), JAMM/MPN domain-associated metallopeptidases (JAMMs), and monocyte chemotactic protein-induced proteins (MCPIPs) [8, 25–27]. These enzymes directly bind to different types of ubiquitin chains and remove ubiquitin chains from the substrates. Engineered deubiquitination synthesis shows that OTUs specifically remove K29-linked ubiquitin chains from the substrate, while JAMMs are often unique to K63-linked ubiquitin chains. Among the DUB subfamilies, the USPs are highly diversified and comprise more than 50 members, forming the largest subfamily of DUBs. USPs undergo mutations in multiple biological processes and are frequently altered in cancers [28, 29] (Fig. 1).

## Structure of DUB3/USP17

**Fig. 2:**
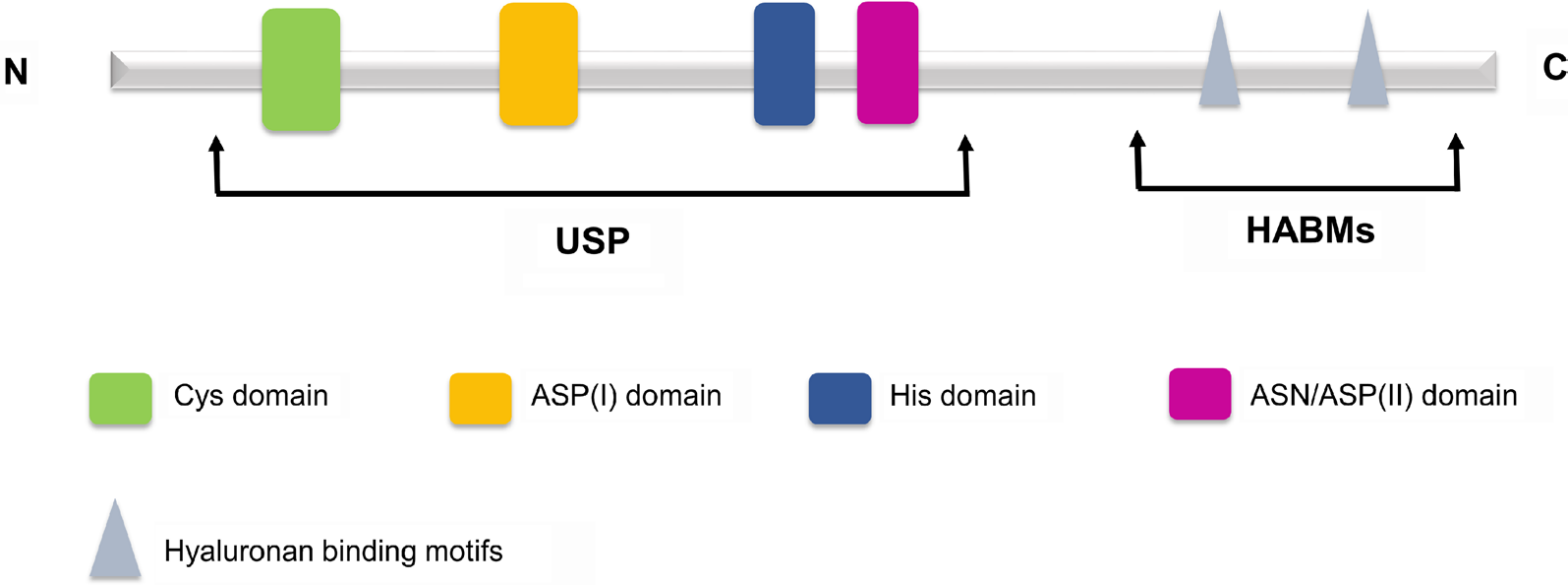
The structure of DUB3/USP17. The USP domain [Cys, Asp (I), His, Asp/Asn (II)] is located at the N-terminus, and the two HABMs are located at the C-terminus

The DUB3/USP17 genes are mainly located on chromosome 4 (4p16.1) and partly on chromosome 8 (8p23.1). According to their sequence homology, human USP17 is divided into 13 subfamily members (USP17A-N) [30], which are encoded by the human mega satellite tandem repeat sequence (RS447) and have unique expression patterns [31]. USP17 contains a USP domain and two hyaluronan (and RNA) binding motifs (HABMs) (Fig. 2). The USP domain is located near the N-terminus, and HABMs are located at amino acids 401–409 and 445–453 near the C-terminus. Each USP domain contains a catalytic component [Cys, Asp (I), His, and Asp/Asn (II)] and is responsible for deubiquitination activity [32]. HABMs exist in the C-terminus of USP17 subfamily members, except in USP17N. HABMs participate in the interaction between USP17 and hyaluronic acid (HA), which inhibits cell proliferation and anchorage-independent tumor growth [33]. In addition, the C-terminal domain also has a role in the catalytic process of USP17, and its deletion significantly reduces the activity of USP17.

## Role of DUB3/USP17 in cancer

### Central nervous system cancer

Glioma is one of the most common tumors in the central nervous system [34]. The expression level of USP17 in glioma tissues is lower than that in normal brain tissues. Forced expression of USP17 inhibits the carcinogenesis and proliferation ability of glioma cells by reducing the protein levels of Ras and Myc. An in vivo orthotopic model has also shown that overexpression of USP17 inhibits glioma progression. A cohort study of 104 patients with glioma has revealed that the expression level of USP17 is negatively correlated with WHO grade [35]. Another study [36] has reported that DUB3 and WEE1, as downstream regulators of the phosphatase and tensin homolog (PTEN)/protein kinase B (Akt) pathway, negatively regulate cell cycle progression and proliferation of glioma cells through cell division cycle 25A (CDC25A). These findings suggest that USP17 exerts a tumor suppressor role in glioma.

### Head and neck cancer

DUB3 is overexpressed in oral squamous cell carcinoma (OSCC) tissues and cell lines, and it has a negative effect on the survival of patients with OSCC. Mechanistically, DUB3 promotes the proliferation of OSCC cells and inhibits apoptosis by triggering the expression of enhancer of zeste homolog-2 (EZH2) by suppressing ubiquitin-mediated bromodomain-containing protein 4 (BRD4) degradation [37].

The tumor suppressor role of the long noncoding RNA, LINC02487, has been demonstrated in OSCC. Silencing LINC02487 leads to the activation of migration and mesenchymal characteristics of OSCC. However, these effects are rescued by downregulating USP17. LINC02487 directly binds to USP17 and negatively regulates USP17. As a downstream molecule of LINC02487, USP17 further exerts its role in epithelial mesenchymal transition (EMT) through posttranslational deubiquitination and stabilization of Snail1 [38]. These studies suggest that DUB3 may be an effective anticancer target for OSCC.

### Thoracic cancer

Many studies have investigated the expression level of USP17 in lung cancer tissues and its clinical significance. McFarlane et al. [39] reported that USP17 is overexpressed in both lung squamous cell carcinoma and adenocarcinoma. However, the expression level of USP17 in squamous cell carcinoma is higher than that in adenocarcinoma. Moreover, the relapse-free survival rate of patients with USP17-positive cancer is significantly lower than that of USP17-negative cancer [39].

Increasing evidence shows that DUBs are involved in the regulation of the cell cycle [40]. It has been reported that deletion of DUB3 blocks the cell cycle of non-small cell lung cancer (NSCLC) cells from G1 to S phase. Mechanistically, DUB3 drives cell cycle progression and promotes lung cancer cell proliferation by deubiquitinating and stabilizing cyclin A [41].

USP17 expression is induced by epidermal growth factor (EGF) stimulation, which is necessary for EGFR endocytosis [42]. Deletion of USP17 blocks the internalization of transferrin and its receptor (TfR). Because TfR is the archetypal substrate for clathrin-mediated endocytosis (CME), silencing USP17 impedes the localization of CME machinery components to the plasma membrane. The impact of USP17 depletion on growth, EGFR endocytosis, and signal transduction has been evaluated in NSCLC cells. For NSCLC cells carrying EGFR activation mutations, deletion of USP17 inhibits the growth of these cells and triggers apoptosis. In addition, deletion of USP17 blocks CME in these cells, but it has no effect on the endocytosis of EGFR mutants and downstream signal transduction. For EGFR wild-type NSCLC cells, suppression of USP17 enhances the sensitivity of the EGFR tyrosine kinase inhibitor (TKI), gefitinib [43]. These data indicate that targeting USP17 is an interesting complement to EGFR TKIs to prevent drug resistance or an alternative after drug resistance is established.

Macrophages are involved in the interaction between inflammation and lung cancer [44]. A connection among USP17 expression, macrophage accumulation, and inflammation has been reported in lung cancer [45]. USP17 is related to the expression level of inflammatory mediators, macrophage markers, and poor prognosis of lung cancer. Tumor necrosis factor receptor-associated factors (TRAFs) are important regulators of inflammatory signaling pathways. The binding motifs of TRAF2 and TRAF3 have been identified in USP17. USP17 removes the K63-linked ubiquitination of TRAF2 and TRAF3, which disrupts the TRAF2/TRAF3 complex. Thus, the target proteins of the TRAF2/TRAF3 complex, namely, NF-kappaB-inducing kinase (NIK), c-Rel, and interferon regulatory factor 5 (IRF5), are activated, which upregulates inflammation-related genes and stemness-related genes in cancer cells [45]. These findings suggest that USP17 drives positive feedback of macrophages and cancer cells to enhance the inflammation and stemness of cancer cells as well as to promote the progression of lung cancer.

Hyaluronan is a type of glycosaminoglycan in the extracellular matrix, and its level is regulated by both hyaluronan-synthesizing enzymes and -degrading enzymes. Due to the uncontrolled expression of the hyaluronan synthase 2 (HAS2) gene and/or changes in HAS2 activity, hyaluronan can be deposited in rapidly remodeled tissues [46]. Hyaluronan synthesized by abnormal HAS2 is closely related to the progression of solid tumors. Compared to normal tissues, the expression level of hyaluronan in lung cancer patients is significantly upregulated, and the elevated level of hyaluronan is regulated by USP17 through deconjugating polyubiquitin chains from HAS2 [47].

Additionally, Zhang et al. demonstrated that suppression of USP17 inhibits NSCLC tumorigenesis and growth by targeting matrix metallopeptidase 3 (MMP3) and MMP9 [48]. Zhang et al. [49] demonstrated that the viability of USP17-overexpressing cells treated with cisplatin is significantly higher than that of control cells. The activation of phosphoinositide-3-kinase (PI3K)/Akt signaling may be one of the contributors to USP17-mediated cisplatin resistance in NSCLC cells. Based on these results, USP17 may be an attractive target for NSCLC management.

### Breast cancer

Lin et al. [50] showed that the expression of USP17 is downregulated in breast cancer tissues and breast cancer cell lines, and they reported that USP17 overexpression significantly inhibits the growth of breast cancer in vitro and in vivo. Further studies have demonstrated that the anticancer function of USP17 is related to asparaginyl endopeptidase (AEP). The ubiquitination of AEP is regulated by TRAF6, which is an E3 ligase that mediates the binding of the K63-linked polyubiquitin chain to proteins. However, the ubiquitination of AEP is reversed by USP17, which subsequently forms a complex with heat shock protein 90α (HSP90α), thus promoting the intracellular stability and secretion of pro-AEP. Blocking the interaction between pro-AEP and TRAF6 or inhibiting HSP90α reduces the secretion of pro-AEP, thus preventing breast cancer metastasis. In addition, USP17-mediated downregulation of AEP protein levels inactivates Erk signaling and inhibits the tumorigenesis and growth of breast cancer [51].

SET domain-containing protein 8 (SET8) is an enzyme that catalyzes the monomethylation of H4K20 (H4K20me1) and participates in tumorigenesis [52]. USP17 is the deubiquitinase of SET8, which interacts with SET8 to remove polyubiquitin chains, thereby stabilizing SET8. Knockdown of USP17 leads to a decrease in SET8 protein levels, which subsequently induces p21 by suppressing H4K20me1 on p21. The induction of p21 then triggers cell senescence and prevents the proliferation of breast cancer cells [53].

Recent research has suggested that DUB3 is involved in the metastasis of triple-negative breast cancer (TNBC). In TNBC, cyclin-dependent kinases 4 and 6 (CDK4/6) activate DUB3 by phosphorylating the Ser41 site of DUB3. Activated DUB3 deubiquitinates and stabilizes Snail1, which is an important factor in promoting EMT and breast cancer metastasis. Therefore, CDK4/6 inhibitors induce DUB3 inactivation, promote Snail1 protein instability, and reduce cell migration, thus inhibiting metastasis in xenograft models of breast cancer [54]. The significance of the CDK4/6-DUB3 axis in regulating breast cancer metastasis has been confirmed by another study [55], demonstrating that the inhibitor of DUB3, WP1130, suppresses DUB3-mediated Snail1 stabilization and that IL-6 stabilizes Snail1 by activating DUB3. In addition to Snail, Slug and Twist are also regulated by the IL-6-DUB3 axis [56]. In basal-like breast cancer (BLBC) cells, IL-6 promotes metastasis by activating DUB3. Activated DUB3 further interacts with Slug and Twist to prevent the degradation of Slug and Twist by deubiquitination, thus promoting the migration, invasion, and cancer stem cell-like characteristics of BLBC cells. Therefore, DUB3 inhibitors significantly inhibit not only the upregulation of Slug and Twist induced by IL-6 in BLBC cells but also the invasion induced by IL-6. These data reveal the role of the IL-6-DUB3 signaling axis in the regulation of EMT progression, suggesting the potential of DUB3 as an anticancer drug target.

CDC25A is the key molecule that promotes oncogenic transformation [57]. It has been reported that DUB3 removes the polyubiquitin modification of CDC25A, which prevents its degradation, thus amplifying the oncogenic role of CDC25A in breast cancer. Knockdown of the DUB3 gene leads to cell cycle arrest in G1/S and G2/M phases. In contrast, overexpression of DUB3 promotes cell accumulation in the S and G2 phases, which is crucial for the oncogenic function of CDC25A [58]. In addition, the impaired activation of GTPases in USP17-depleted cells causes the accumulation of the cyclin-dependent kinase inhibitors (CDKIs) p21^cip1^ and p27^kip1^ and G1 phase arrest [59].

Geminin is an inhibitor of chromatin licensing and DNA replication factor 1 (Cdt1) [60], and DUB3 regulates Geminin through deubiquitination. DUB3 overexpression increases the level of Geminin and is associated with genomic instability, DNA replication change, aneuploidy, and cancer progression [61]. These data confirm that DUB3 regulates DNA replication by controlling Geminin levels, thereby suggesting a new role of DUB3 in breast cancer progression.

In breast cancer, the combined use of bromodomain and extraterminal domain (BET) inhibitors with histone deacetylase (HDAC) inhibitors upregulates the expression level of USP17. The increased expression of USP17 leads to a reduction in the Ras/mitogen-activated protein kinase (MAPK) signaling pathway and cell viability, while siRNA-mediated USP17 silencing significantly reverses the cytotoxicity of the combined treatment of BET and HDAC inhibitors [62].

Pristimerin (20α-3-hydroxy-2-oxo-24-nor-friedela-1-10,3,5,7-tetraen-carboxylic acid-29-methyl ester) is a natural quinone methide triterpenoid isolated from Celastraceae and Hippocrateaceae. Studies have shown that pristimerin has insecticidal, anti-inflammatory, antiangiogenic, antiprotozoal, and anticancer effects [63]. A recent study has found that pristimerin has an anticancer effect on breast cancer [64]. Pristimerin inhibits breast cancer progression by upregulating miR-542-5p. MiR-542-5p binds to Argonaute 2 (AGO2) and inhibits the expression of DUB3 in breast cancer cells. These findings increase the understanding of the upstream regulator of DUB3.

### Gastrointestinal cancer

Zinc finger E-box combined with homeobox 1 (ZEB1) is an important inducer of chemotherapy and radiotherapy resistance in cancers [65]. USP17 has been identified as the downstream regulator of ZEB1 in colorectal cancer cells [66]. H2A histone family member X (H2AX) is required for the recruitment of proteins involved in DNA repair [67]. As a deubiquitylating enzyme, USP17 inhibits the DNA damage-induced ubiquitination of H2A histone family member X (H2AX) [68], which may contribute to ZEB1-mediated chemotherapy resistance [66]. In addition, by directly controlling the level of H2AX deubiquitination, USP17/DUB3 regulates recruitment of the DNA repair factors p53-binding protein 1 (53BP1) and breast cancer susceptibility gene 1 (BRCA1), in DNA damage sites to ensure a correct DNA damage response (DDR) [68]. USP17 is also involved in the repair of DNA damage by mediating defective chorion-1 (DEC1), a molecule that plays a key role in DNA damage repair. To cope with DNA damage caused by genotoxic stress, USP17 activates and stabilizes DEC1 through deubiquitination. Subsequently, DEC1 is degraded by SCF^βTrCP^ ubiquitin ligase and CK1α on the proteasome, which is necessary for effective recovery from the G2 DNA damage checkpoint [69].

Finally, it has been reported that DUB3 leads to chemotherapy resistance through Nrf2 deubiquitination and stabilization [70]. The interaction between DUB3 and Nrf2 removes the K48-linked polyubiquitination of Nrf2, which ultimately inhibits the degradation of Nrf2 and allows formation of a functional complex of Nrf2 and Keap1, resulting in DUB3-induced chemotherapy resistance.

### Genitourinary cancer

Prostate cancer is one of the most common male cancers in the world [71]. USP17 is highly expressed in prostate cancer tissues and cell lines. Prostate cancer patients with low expression levels of USP17 have a better overall survival rate. Inhibition of USP17 expression significantly induces apoptosis and downregulates the proliferation, migration, and invasion of prostate cancer cells. The anticancer effect of USP17 has been verified in a subcutaneous mouse model. Mechanistically, inhibition of USP17 blocks NF-κB signal transduction by promoting reactive oxygen species (ROS) production [72].

DUB3 binds to BRD4 to promote its deubiquitination and stabilization in prostate cancer. The sensitivity of prostate cancer cells to the BET inhibitor, JQ1, depends on the deubiquitinating effect of DUB3 on BRD4. Therefore, DUB3 inhibitors may promote the degradation of BRD4 and reverse the drug resistance effect of JQ1 in prostate cancer. DUB3 itself is transcriptionally repressed by the nuclear receptor corepressor 2 (NCOR2)-HDAC10 complex. NCOR2 deletion leading to activation of DUB3 and BRD4 protein is often detected in castrated prostate cancer patients. These findings suggest that DUB3 leads to drug resistance to BET inhibitors by stabilizing the BRD4 protein, suggesting that DUB3 is a feasible therapeutic target for overcoming the drug resistance of BET inhibitors in prostate cancer [73].

### Gynecologic cancer

It has been demonstrated that overexpression of USP17 significantly induces apoptosis and inhibits cell proliferation in cervical adenocarcinoma cells. Further study has revealed that the inhibitory effect of USP17 depends on suppressor of defective silencing 3 (SDS3)-related HDAC activity. The C-terminus of USP17 binds to the N-terminus of SDS3 and colocalizes in the nucleus, specifically deubiquitinating the K63-linked polyubiquitin chains of SDS3, which alters SDS3-associated HDAC activity [74]. However, deletion of HABMs in USP17 blocks the interaction between USP17 and SDS3 but retains the deubiquitinating activity of USP17 toward SDS3 [75].

Ras mutations, including H-Ras, N-Ras, and K-Ras, are common events that trigger carcinogenesis, and up to 30% of human cancers have these types of mutation [76]. It has been reported that USP17 inhibits the localization of H-Ras and N-Ras to the plasma membrane while not affecting the localization of K-Ras4b. In USP17-overexpressing cells, abundant N-Ras has been demonstrated to be located in the endoplasmic reticulum and Golgi. Abnormal localization of Ras induced by USP17 leads to downregulation of the MAPK, Mek/Erk, and PI3K/JNK signaling pathways, resulting in delayed growth of HeLa cells [77]. In addition, USP17 also deubiquitinates Ras converting enzyme 1 (RCE1), which is important for the activation of Ras. USP17 negatively regulates the activity of RCE1 by removing the K63-linked polyubiquitin chains conjugated to RCE1 [78]. These findings suggest that USP17 regulates differential Ras isoform signaling from different intracellular platforms, making it an important protein for further research related to potential cancer therapy.

ETS-like protein 1 (ELK-1) is a transcription factor that participates in Erk-induced cell proliferation [79]. The transcriptional activity of ELK-1 is regulated by ubiquitination at lysine 35. As a DUB, USP17 reverses the ubiquitination of ELK-1 and elevates the expression level of ELK-1-targeted genes, which is accompanied by activation of Cyclin D1. In contrast, the deletion of USP17 suppresses the expression level of ELK-1 target genes and inhibits cell proliferation [80]. This evidence suggests that USP17 is also involved in the regulation of cervical cancer cell proliferation through mediating the ELK-1 signaling pathway.

USP17 has a predominant role in regulating cell migration and cytoskeletal reorganization. In USP17-deficient cells, the protrusion decreases, the shape becomes round, and cytoskeletal polymerization is reduced, and the migration distance and migration rate significantly decrease. USP17 is induced by the stromal cell-derived factor-1 (SDF-1)/C-X-C motif chemokine ligand 12 (CXCL12) and IL-8/CXCL8 chemokines in cancer cells. However, the absence of USP17 hinders normal cytoskeletal rearrangement and chemokine-induced membrane localization of Rho GTPases, including CDC42, Rac, and RhoA, which are essential for cell motility [81]. These results suggest that USP17 is necessary for GTPase subcellular localization and cell motility, indicating that it may be a useful drug target for the treatment of cancer metastasis.

Ovarian cancer is the main cause of death of gynecological malignancies [82]. Immunohistochemistry analysis has shown that the expression level of DUB3 in ovarian cancer tissues is higher than that in normal ovarian tissues. The expression level of DUB3 is closely related to lymph node metastasis, advanced clinical stage, and poor prognosis. A previous in vitro study has shown that DUB3 silencing induces apoptosis and inhibits the proliferation of ovarian cancer cells by arresting the cell cycle in G0/G1 phase [83].

Chemotherapy is an important component of the treatment for ovarian cancer [84]. Myeloid cell leukemia sequence 1 (MCL1) plays a critical role in the regulation of chemoresistance in ovarian cancer. In ovarian cancer cells, DUB3 interacts with MCL1 to deubiquitinate and stabilize MCL1 through its 40th lysine at the N-terminus. In addition, *O*6-methylguanine-DNA methyltransferase (MGMT) is a key activator of DUB3 transcription. The MGMT inhibitor, PaTrin-2, effectively inhibits DUB3 at the transcriptional level. Therefore, DUB3 suppression or PaTrin-2 treatment significantly induces apoptosis of ovarian cancer cells by downregulating MCL1. Furthermore, the expression of MGMT/DUB3 is activated by HDACis. A synergetic therapeutic effect has been shown with the combined use of HDACis and PaTrin-2. These results suggest that the MGMT-DUB3-MCL1 signaling axis has a role in the regulation of chemoresistance in ovarian cancer [85].

### Bone and soft tissue sarcomas

USP17 exerts an oncogenic role in osteosarcoma. The expression of USP17 is upregulated in osteosarcoma tissues, MG-63 cells, and U2OS cells. Functional experiments have shown that USP17 promotes the proliferation, migration, and invasion of osteosarcoma cells. USP17 interacts with Smad4 to stabilize Smad4 through its DUB activity and then promotes EMT to enhance the migration and invasion of osteosarcoma cells [86].

## Conclusions

**Fig. 3:**
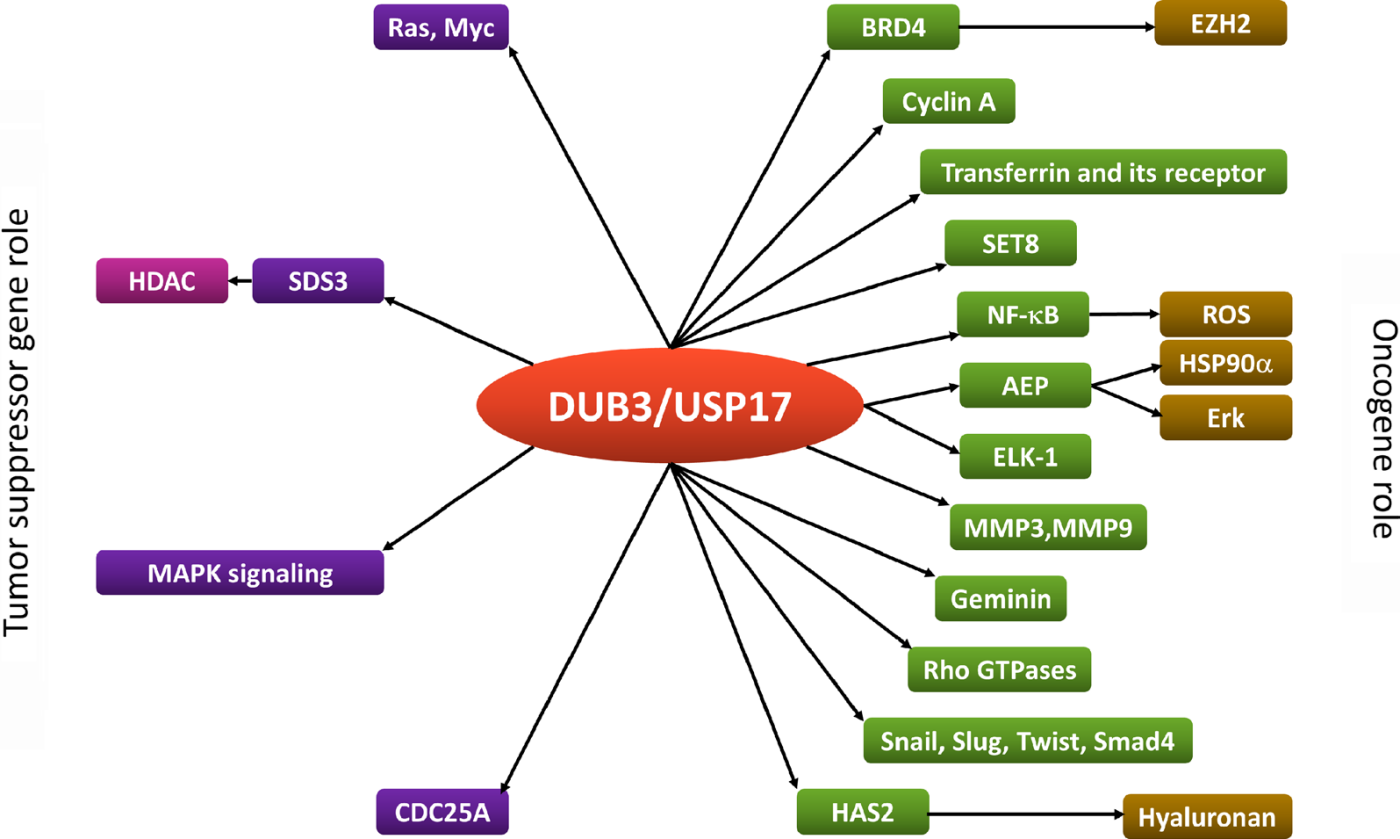
The oncogenic and the tumor suppressor role of DUB3/USP17 in cancer and the pathways in which it participate

In the past decade, DUBs have become attractive targets for cancer treatment. As an important DUB, DUB3/USP17 is involved in the regulation of most, if not all, cancer hallmarks, especially the signal transduction pathways that confer cell cycle progression, proliferation, apoptosis, and treatment resistance (Table 1). In this review, we summarized the oncogenic and the tumor suppressor role of DUB3/USP17 in cancer (Fig. 3). However, most of the recent studies have focused on the function of USP17 in regulating protein degradation and stability. In addition to participating in protein ubiquitination, the nonubiquitin function of USP17 also needs to be further studied in cancer. Furthermore, it is necessary to clarify how USP17 achieves specificity through substrate interactions in different tumor microenvironments because recent studies have shown that USP17 regulates the stability and nuclear function of IL-33 [87]. Finally, more data on USP17 may lead to the development of specific USP17 inhibitors/agonists for cancer treatment. Discovery of the role of USP17 in cancer may provide valuable information to prevent carcinogenesis and develop effective drugs.

**Table 1:** The roles of USP17 in different types of cancers

Cancer type	Targets	Functions	References
Central nervous system cancer	Ras and Myc	Inhibits the carcinogenesis and proliferation of glioma	[35]
	CDC25A	Negatively regulates cell cycle progression and proliferation of glioma	[36]
Head and neck cancer	BRD4/EZH2	Promotes the proliferation of OSCC cells and inhibits apoptosis	[37]
	Snail1	Promotes the epithelial mesenchymal transition (EMT)	[38]
Thoracic cancer	Cyclin A	Drives cell cycle progression and promotes lung cancer cell proliferation	[41]
	Transferrin and its receptor (TfR)	Promotes the growth of EGFR mutation lung cancer cells and inhibits apoptosis	[43]
	TRAF2 and TRAF3	Upregulates the inflammation-related genes and stemness-related genes in cancer cells	[45]
	HAS2	Triggers the progression of cancer	[47]
	MMP3 and MMP9	Promotes the tumorigenesis and growth	[48]
	PI3K/Akt	Contributes to cisplatin resistance in NSCLC cells	[49]
Breast cancer	AEP	Promotes the intracellular stability and metastasis	[50]
	SET8	Increases the proliferation of breast cancer cells	[53]
	Snail, Slug and Twist	Promotes EMT and breast cancer metastasis; cancer stem cell-like characteristics of breast cancer cells	[54, 56]
	CDC25A	Promotes cell cycle progression	[58]
	Geminin	Enhances genomic instability, DNA replication change, aneuploidy, and cancer progression	[61]
	MAPK signaling pathway	Decreases the cell viability	[62]
Gastrointestinal cancer	H2AX	Overcomes DNA double-strand break (DSB)	[66, 68]
	DEC1	Recovers from G2 DNA damage checkpoint	[69]
	Nrf2	Leads to chemotherapy resistance	[70]
Genitourinary cancer	NF-κB/ROS	Promotes the proliferation, migration, and invasion of prostate cancer cells and inhibits apoptosis	[72]
	BRD4	Leads to drug resistance of BET inhibitor	[73]
Gynecologic cancer	SDS3	Induces apoptosis and inhibits cell proliferation in cervical adenocarcinoma cells	[74]
	Ras	Decreases growth of Hela cells	[77]
	ELK-1	Promotes cell proliferation	[80]
	Rho GTPases	Promotes cell migration and cytoskeleton reorganization	[81]
	MCL1	Regulates chemoresistance in ovarian cancer	[85]
Bone and soft tissue sarcomas	Smad4	Enhances the migration and invasion of osteosarcoma cells through activation of EMT	[86]

*AEP* asparaginyl endopeptidase, *Akt* protein kinase B, *BET* bromodomain and extra-terminal domain, *BRD4* bromodomain-containing protein 4, *CDC25A* cell division cycle 25A, *DEC1* defective chorion-1, *EGFR* epidermal growth factor receptor, *ELK-1* ETS-like protein 1, *EZH2* enhancer of zeste homolog-2, *H2AX* H2A histone family member X, *HAS2* hyaluronan synthase 2, *MAPK* mitogen-activated protein kinase, *MCL1* myeloid cell leukemia sequence 1, *MMP3* matrix metallopeptidase 3, *OSCC* oral squamous cell carcinoma, *PI3K* phosphoinositide-3-kinase, *ROS* reactive oxygen species, *SDS3* suppressor of defective silencing 3, *SET8* SET domain-containing protein 8, *TRAFs* tumor necrosis factor receptor associated factors

## Data Availability

None.

## Abbreviations

53BP1: P53-binding protein 1
AEP: Asparaginyl endopeptidase
AGO2: Argonaute 2
Akt: Protein kinase B
ATP: Adenosine triphosphate
BET: Bromodomain and extra-terminal domain
BLBC: Basal-like breast cancer
BRCA1: Breast cancer susceptibility gene 1
BRD4: Bromodomain-containing protein 4
CDC25A: Cell division cycle 25A
CDK4/6: Cyclin-dependent kinases 4 and 6
CDKIs: Cyclin-dependent kinase inhibitors
Cdt1: Chromatin licensing and DNA replication factor 1
CME: Clathrin mediated endocytosis
CXCL12: C-X-C motif chemokine ligand 12
DDR: DNA damage response
DEC1: Defective chorion-1
DSB: DNA double-strand break
DUBs: Deubiquitinating enzymes
EGF: Epidermal growth factor
EMT: Epithelial mesenchymal transition
EZH2: Enhancer of zeste homolog-2
H2AX: H2A histone family member X
HABMs: Hyaluronan (and RNA) binding motifs
HAS2: Hyaluronan synthase 2
HDAC: Histone deacetylase
HECT: Homologous to the E6-AP carboxyl terminus
HR: Homologous recombination
HSP90α: Heat shock protein 90α
IL-4: Interleukin-4
IRF5: Interferon regulatory factor 5
JAMMs: JAMM/MPN domain-associated metallopeptidases
MAPK: Mitogen-activated protein kinase
MCL1: Myeloid cell leukemia sequence 1
MCPIPs: Monocyte chemotactic protein-induced proteins
MGMT: *O*6-Methylguanine-DNA methyltransferase
MJDs: Machado–Joseph (Josephin) domain proteases
MMP3: Matrix metallopeptidase 3
NCOR2: Nuclear receptor corepressor 2
NHEJ: Non-homologous end joining
NIK: NF-kappaB-inducing kinase
NSCLC: Non-small cell lung cancer
OSCC: Oral squamous cell carcinoma
OTUs: Ovarian tumor proteases
PI3K: Phosphoinositide-3-kinase
PTEN: Phosphatase and tensin homologue
PTMs: Post-translational modifications
RBR: Ring between ring fingers
RCE1: Ras converting enzyme 1
RING: Really interesting new gene
ROS: Reactive oxygen species
SDF-1: Stromal cell-derived factor-1
SDS3: Suppressor of defective silencing 3
SET8: SET domain-containing protein 8
TfR: Transferrin and its receptor
Th17: T helper 17 cells
TKI: Tyrosine kinase inhibitor
TNBC: Triple negative breast cancer
TRAFs: Tumor necrosis factor receptor associated factors
UCHs: Ubiquitin COOH terminal hydrolases
USP17: Ubiquitin-specific protease 17
ZEB1: Zinc finger E-box combined with homeobox 1
